# Hospital and Institutional News

**Published:** 1915-06-12

**Authors:** 


### HOSPITAL AND INSTITUTIONAL NEWS.

### THE BIRTHDAY HONOURS.

The Honours List this year, which appeared on the King's fiftieth birthday, is far larger than usual by reason of the attempts made to include in it those whose services to the nation have been especially noteworthy during the progress of the war. While we all know that the medical services, including hospital work, have been of the most invaluable aid, and come through the present war with more credit than in any previous conflict, we must not expect to see these workers included so much in the Honours List as in the periodical lists of distinctions for military service which are issued at intervals. From the normal institutional standpoint the most noticeable distinction in the Honours List is the knighthood bestowed on Mr. C. S. Loch, the recently retired and veteran Secretary of the Charity Organisation Society. It is interesting to recall that before he took up this post in 1875 he had been for two years clerk to the Royal College of Surgeons. In 1893-5 he was a member of the Royal Commission on the Aged Poor, and later of the Royal Commission on the Care and Control of the Feeble-minded. He also served, of course, on the Poor-Law Commission. Two other notable knighthoods are those given to Mr. James Mackenzie, M.D., F.R.C.P., Lecturer on Cardiac Research at the London Hospital, and Mr. Frederick Needham, one of the Commissioners of the Board of Control, who had been one of the Lunacy Commissioners since 1892. In the Military Division of the Order of the Bath, Surgeon-General Thomas Martin Corker, M.D., K.H.P., receives the C.B., while we are glad to record that Mr. Arthur William Garrard Bagshawe, M.B., Director of the Tropical Diseases Bureau, is honoured by the C.M.G. The distinction of Knight Bachelor is given to Mr. Charles Frederick Fraser, LL.D., Superintendent of the School for the Blind, Halifax, Canada.

### HONOURS FOR ACTIVE SERVICE.

Among those serving at the Dardanelles who are also honoured are Major E. J. O'Neill, M.B., F.R.C.S., New Zealand Medical Corps, and Capt. A. G. Butler, Australian Army Medical Corps, both of whom receive the D.S.O. The Distinguished Conduct Medal is given to No. 1065 Staff Sergeant H. Jackson, Australian Army Medical Corps; No. 3/95 Lance-Corporal W. Singleton, New Zealand Field Ambulance; and No. 3/447 Lance-Corporal G. Stedman, New Zealand Medical Corps, for devotion to duty while serving at the Dardanelles.

### THE INDIAN LIST.

In the Indian List Lieut.-Colonel Cecil Charles Stewart Barry, Indian Medical Service, Medical Superintendent General Hospital, Rangoon, Burma, receives the C.I.E.; the Kaisar-i-Hind Gold Medal is bestowed on Lady Lukis, the wife of Surgeon-General Sir C. P. Lukis, K.C.S.I., Director-General, Indian Medical Service, and Dr. Thomas Franklin Pedley, M.D., V.D., medical practitioner, Rangoon, Burma, and Surgeon Lieut.-Colonel in the Rangoon Port Defence Volunteers. Also the Imperial Service Order is given to Rai Chuni Lal Basu Bahadur, M.B., F.C.S., First Assistant Chemical Examiner of Government, Teacher of Physics and Chemistry, Campbell Medical School, and Fellow of the Calcutta University; and to James Edward Aquart Ferguson, Esq., M.B., Government Medical Officer, Colony of British Guiana.

### WAR SERVICE BADGES FOR HOSPITAL STAFFS.

The industry of the amateur recruiting sergeant, male and female, has grown to such an extent that not only are individual hospitals either issuing Special Service badges to be worn by members—particularly, of course, the young members—of their staffs, but the British Hospitals Association has been approached for advice and assistance on the matter. We understand the view of the Association to be that the matter is hardly one on which its action is necessary, but rather that the question should be decided in each locality where the hospital staffs are subjected to cross-questioning and ridicule on their way to and from the hospital where they are patriotically at work. Readers of The Hospital will have noted in our account of the latest developments at the London Hospital last week that a War Service badge has been issued by that institution. In our Bureau of Information last week there also appeared a question on the subject. In view of the uncertainty which prevails, we hope next week to publish an article on the point, in which the practice in different institutions will be discussed, badges in use illustrated, and the problem of issuing them in the case of the smaller hospitals dealt with. In this way a practical solution will be given to an apparently small matter, which, however, is exercising many hospital managers and their staffs just now.

### THE DISMISSAL OF REFRACTORY SANATORIUM PATIENTS.

The right of a medical officer to discharge insured patients from a sanatorium for breaches of the regulations has been lately discussed by the Huddersfield Insurance Committee. It appears that the medical officer, Dr. Moore, discharged three patients, two of whom were observed by nurses on the road out of bounds, while the third was discharged for refusing to take exercise. The third man had been discharged for declining to join in the trenching which was being carried on by the other patients in the hospital grounds; but on being cross-examined by a member of the Committee Dr. Moore declined to admit that he was discharged for refusing to work, except in the sense that exercise could not be distinguished from it. The Chairman of the Committee said that he could find no rule which justified the dismissal of a patient. The question was then raised as to whether a patient had any redress, and, if so, to whom he should appeal. The man, said the speaker, had paid for his treatment, and had a right to have it. This, no doubt, is true; but no right is unconditional, and one of the conditions attaching to efficient sanatorium treatment is the exact obedience of the patients to the necessarily precise *régime* prescribed by the medical superintendent. It has always been found essential to sanatorium treatment that precise regulation of rest times and times of exercise should be made; and, rather than these should be interfered with, the medical superintendent has always assumed the right to dismiss patients who do not carry out the prescribed treatment. Sanatorium treatment more than any other depends on discipline, and though no wise medical superintendent will resort to dismissal except as a last resource, yet power to dismiss patients is necessary to the efficient discharge of his duties. The Huddersfield Insurance Committee's only concern is whether Dr. Moore's action was judicious; not whether it was allowable.

### AIR RAIDS ON HOSPITALS.

In these days of air raids the lot of administrators and those responsible for the hospitals in the areas affected is not an enviable one. The feeling of utter helplessness not only applies to the patients, but is equally shared by all, for apparently nothing can be done except to make sure that all appliances for combating outbreaks of fire are in working order, the staff familiar with the use of them, and, when the word is given, to "stand by" ready for immediate action. It is, indeed, a time for discipline and cool heads, for great demands may be made upon them, as was the case of a civil hospital hit by an incendiary bomb the other day. It fortunately fell through the administrative block, so there was no loss of life, nor were the patients endangered. The authorities of the hospital in question are proud to state that the whole of the staff behaved with promptitude and calmness. Some attacked the flames and took the necessary precautions until the arrival of the fire-brigade, while others admitted and attended casualties from fires and falling houses. Is roof-protection possible?

### A GERMAN MATRON.

So far, the voluntary hospitals have been spared the bitter controversy that has ensued in other establishments through the presence on the staffs of persons of German parentage or nationality. It is therefore with regret that we refer to the recent controversy which has occurred over the desirability or otherwise of retaining the services of Miss H. Rettberg, matron of the Devizes Cottage Hospital. The facts appear to be as follows. One of the subscribers has protested against the employment of Miss Rettberg as matron on the ground that she is a German. No one denies, it seems, that she is of German birth, and resided in that country during the first two decades of her life or thereabouts. Objectors, therefore, describe her as "an enemy alien," who should not be in charge of a public institution in this country. The controversy has been taken up vigorously in the local Press, but the only letter which it would serve a useful purpose to quote is that which appeared in the *Wiltshire Gazette* of June 3, signed by four members of the hospital's medical staff. From that we quote the following passage: "We are of opinion that the members of the committee who elected Miss Rettberg, and who are acquainted with her views and antecedents, are the proper authority to decide on this matter, and so long as they, with the knowledge they possess, are satisfied that they are doing their duty to the hospital and the country in retaining her we think the public should respect their judgment." The writers also are at pains to emphasise Miss Rettberg's loyalty to England, and urge that the subscribers should come to no "hasty and ill-considered decision." In our view, the matter has gone far enough to make expedient the putting into operation of that rule of the institution by which, we understand, any six subscribers may call a general meeting. If the feeling of the public, which within proper limits it is the duty of the committee to respect, is really opposed on general grounds to the retention of the present matron, the committee's personal belief in her must give way to the wish of those whom they are appointed to represent. The best test of public feeling would be the calling of a general meeting in due form, as indicated by the rules, in which the objectors and the committee would be able to present their cases and to act on the vote of the subscribers. If the matter is not settled in proper form nothing but harm can accrue to the hospital from the controversy.

### TRAVELLING SCHOOL HOSPITALS AND CLINICS.

The war has made this the age of mobile operating theatres, motor ambulances, and institutions on wheels, and therefore the announcement that Australia has lately inaugurated a travelling school hospital excites less surprise than would have been the case a year ago. The hospital visits those parts of the country where there are no resident medical men or dentists, and the children at the remote schools receive a medical examination. Spending a few days at each centre the hospital, whose staff consists of two medical officers, a dental surgeon, and a nurse, in four months dealt with 2,558 child patients. Sufficient success led to the establishment of a travelling ophthalmic clinic, which, like the travelling school hospital, is the latest outcome of the system of State education in Australia. It is interesting to compare in this connection the temporary buildings for dispensaries and clinics in rural districts, one of which was described in The Hospital a week or two ago.

### THE SOUTH LONDON HOSPITAL FOR WOMEN.

The Marchioness of Londonderry had some encouraging things to say as to the progress of the new South London Hospital for Women, now in course of erection at Clapham Common, at the third annual meeting of the subscribers held at Sunderland House, Curzon Street, W., by kind permission of the Duchess of Marlborough. The board of management have been able to make arrangements with the nursing home for the use of a large room containing four beds pending the day when the new hospital will be ready for occupation; in eight months fifty-one patients have received treatment. Three anonymous donations of one hundred guineas each have been contributed for the upkeep of this department, and enabled the board to send to other hospitals women who had come under the care of the out-patients' department. The latter is situated at Newington Causeway, and during the year 4,242 patients made between them 14,650 attendances, the number of patients having doubled those of the previous year. The Duchess of Marlborough said that an analysis of the districts from which the patients came showed that many resided in localities where the greatest destitution was to be found, and that in many cases patients had come long distances thus showing the absolute need for such a hospital conducted by medical women. It has been decided to go on with the erection of the new hospital at Clapham Common with all dispatch, and this can be done owing to a munificent gift of £16,000, though, through the increase in the price of building and other materials due to the war, the Secretary, Miss M. E. Ridley, is understood to estimate that the amount will not cover the entire cost by at least £2,000. It is realised by the short three years' history that there is a growing need in South London for the Hospital for Women, and therefore efforts are to be increased to bring it to an early fruition.

### MEDICAL WOMEN AND MARRIAGE.

The Annual Report of the London (Royal Free Hospital) School of Medicine for Women contains a complete list of registered medical women who were former students of the school. These number 585, and we notice that 133 who were single women at the time of registration have since entered the ranks of the married; but the fact that of the total register only twenty-three have retired from practice since registration shows that marriage has not robbed the profession at large of a number of useful members. The Council has secured the building lease of an adjoining piece of land, upon which it is proposed to erect a large block, containing extensions of the departments of anatomy, physiology, chemistry, and physics, a department for pathological research, and more general accommodation to meet the needs of the increasing number of students. The Council have been in close communication with the Departmental Committee of the University of London appointed by the Board of Education to assist in carrying out the recommendations of the Royal Commission, and presented to them a memorandum stating the claims of the school to be recognised as a constituent college of the Faculty of Medicine in any reconstitution of the University.

### TOOTING HOME AS A MILITARY HOSPITAL.

The War Office has taken over for the use of the wounded and sick soldiers the Tooting Home, which is under the control of the Wandsworth Board of Guardians. Formerly a Roman Catholic school, the property was purchased by the Guardians, wh[illegible] equipped the buildings, which were enlarged, as an institution for over 600 aged and infirm folk. There are large gardens and wide-spreading grounds. The inmates have been removed to Mitcham Workhouse, which belongs to the Holborn Union, and the other institutions under the charge of the Wandsworth Board of Guardians. Many of the old people were loath to leave their old quarters, for Tooting Home was a pleasant place, abounding in comforts, and not a few had lived there for a great many years. The Local Government Board have expressed their appreciation of the patriotic action of the Guardians. So keenly have the inmates felt the severance that one old man, sixty-five years of age, who had been in the Home fourteen years, committed suicide by drowning in the Thames. Others have complained that at Mitcham they have to wear a distinctive dress, on which is conspicuously stamped to whom it belongs, and where it is to be returned in case the wearer casts it aside. After the freedom of Tooting Home, the old folk have felt acutely the changes that mark them as belonging to the poor kept by the rates. Many years ago the Lambeth Board of Guardians discarded clothing marked in this way, and it would naturally have been thought that Holborn had long ago followed their example. The sooner they do so the better for all with whom they are brought into contact; and the fact that those now in their charge have never been accustomed to these labels should make the present an admirable opportunity to abolish them.

### GENERAL MEDICAL COUNCIL'S NEW HOME.

The summer session of the General Medical Council was held last week, and among other matters referred to in the President's Address was the announcement that the Council hoped to enter into occupation of their new quarters by the end of this year. The site of the new offices is in Hallam Street, which runs parallel to Portland Place, between that thoroughfare and Great Portland Street. This position will be a most convenient one for the purpose, probably more suitable than the old site in Oxford Street, which has been sold to advantage. The new buildings will certainly be much more commodious; they have been designed by Mr. Eustace Frere, A.R.I.B.A., whose plans have been accepted for exhibition at the Royal Academy. The contractors are Messrs. Chinchen and Co.

### EX-HOSPITAL CLERK AND OFFICER SENTENCED.

At the Thames Police Court last week Robert P. Macgrath, a lieutenant in the King's Own Royal Lancaster Regiment, was summoned for obtaining £234 19s. 6d. by false pretences from Lord Knutsford. The plaintiffs' case was that the defendant, who was in uniform and wearing the South African war ribbons, after being a clerk at the London Hospital was appointed secretary to Lord Knutsford at a salary of £350 per annum. It was alleged that he obtained money by recording fictitious names as those of persons each of whom had received weekly salaries. In September, having expressed a wish to go to the Front, Lord Knutsford obtained a commission for him, and a sword was presented to him by the staff. He became severely wounded, and lost one arm. After he left the defalcations were discovered. It was stated that while Lord Knutsford wished the case dealt with in the Police Court, he could not recommend him to leniency, as the defendant had obtained other large sums by similar means. On the defendant's behalf it was stated that he had served with distinction in the South African and in the present war, and that his left arm was shattered and his foot injured. The magistrate, who described the case as painful in view of the defendant's services, sentenced him to six months' imprisonment in the second division. The defendant had to be carried out of court.

### HOSPITAL MEMORIAL FOR GRETNA DISASTER.

A meeting of representative citizens of Leith has decided to establish a memorial to the officers, non-commissioned officers, and men of the 7th Royal Scots, who lost their lives in the Gretna railway accident. While it was felt that the day might well come later when an imposing memorial would be erected to the men of Leith who had fallen in the war, that day was not yet come. In the meantime there is a strong wish to place a memorial in the Rosebank Cemetery. In addition, it has been decided to endow a bed or beds in Leith Hospital in memory of the men who died at Gretna.

### THE BELGIAN FIELD HOSPITAL AFFAIR.

In our last issue we quoted the statements which had appeared in the papers explaining the reasons which had led Mr. Harold Hodge to resign the chairmanship of the Belgian Field Hospital. Since then Lord Sydenham, the president of the institution, has also written to the papers, and as he alleges that Mr. Hodge's statements are "exactly calculated to mislead the public," it is only fair to summarise his remarks. Lord Sydenham points out that as the institution is the only British hospital acting under the direction of the Belgian War Department, the Department's instructions that a Belgian surgeon be appointed to the staff left the Committee no alternative but to acquiesce or close the hospital. Lord Sydenham goes on to say that Mr. Hodge, finding himself in a minority of one on the Committee, then resigned; that since the change the King of the Belgians and a French General have expressed their surprise at the efficiency of the arrangements; and that, while the hospital remains "under the direction" of the Belgian authorities, "British control is maintained, as always, by the fact that the subscribed funds are administered in London by the Committee, who also appoint the whole staff, with the single exception" already noted. Lord Sydenham finally concludes by saying that the hospital has not done its work and may at any moment be urgently needed. Readers will notice that while Lord Sydenham says that instructions were received from the Belgian authorities that "a Belgian surgeon should be appointed," Mr. Hodge wrote that these authorities "required that the head of the hospital's medical staff shall be a Belgian, responsible to the Belgian authority and not to the Committee." That is not quite the same thing, and whether Lord Sydenham has glossed over the fact, or Mr. Hodge has exaggerated, the plain fact remains that a committee which raises money but does not have the last word in appointing its officers is in a difficult position. While tact and good sense on both sides may overcome this, sound administrative principles demand that the power of the purse and the power of appointment should be in the same hands. The sort of thing which happens when they are not is exemplified in this correspondence.

### SUGGESTED BASE HOSPITAL FOR BRADFORD.

A meeting presided over by the Lord Mayor of Bradford, Mr. G. H. Robinson, has considered the desirability of establishing a base hospital for wounded soldiers in place of the existing scattered accommodation. At present the wounded, who are mainly convalescent cases sent on from Leeds, are received at the Royal Infirmary, the Eye and Ear Hospital, the Guardians' Bowling Park Colony, and the Fieldhouse Auxiliary Hospital. Some time ago the War Office contemplated establishing a base hospital at Dumb Mills, but the scheme fell through owing to the mills being let. As things are it is felt that the existing possibilities are not made use of to their fullest extent, owing to the decentralised system of accommodation; while if a base hospital were established, it is understood that 500 soldiers could be accommodated and brought straight to the city. A committee has been appointed to consider the proposal, which, if adopted, would have the double effect of keeping all the beds reserved for military patients filled (which they are not at present), and also of allowing other than convalescent cases to be received. At present Bradford receives only those which have been sent from other centres where base hospitals exist.

### THE NATIONALITY OF A GUY'S MEDICAL STUDENT.

Some curious points arose in a case last week in which a medical student of Guy's Hospital was summoned under the Aliens' Restrictions Order at Greenwich Police Court for failing to register as an alien enemy. The defendant, who changed with his family his name in August from Kopelowitz to Kerby, declaring they were British subjects, was born, said the prosecution, in Berlin. His father claimed to have been born in Sweden, but married a German woman, and in 1885 went to Cape Town and became naturalised as a British subject. It was argued for the Home Department that aliens who were naturalised in British possessions were not naturalised in England. The defendant's birth was registered at the British Consulate in Berlin in 1894, and he lived in that city till May 1913. The defendant objected to the use of the word "enemy," and the prosecution only asked that he should be required to register. The defendant said he wished to show his British feelings by fact. He wanted to take part in the war and expected to be called up shortly as a dresser. He argued that he was simply required to furnish particulars. He had not enlisted because the head of the R.A.M.C. had told him that the first duty of medical students was to continue their studies. In Germany "civis Britannicus" was always added to his papers. His Berlin flat was filled with pictures of the King, Lord Kitchener, etc. The National Anthem of the Union of South Africa was composed by one of his brothers.

### THE VALUE OF SMALL FUNCTIONS.

The annual sale of work promoted by the Ladies' Association of the Gravesend Hospital to provide linen and other necessaries for the children's ward was held in the Hospital Garden last week, and resulted in a sum of £77 being raised for the purpose named. The result is very gratifying, since it is £10 more than was raised last year. The band of the Queen's Own 2/5th R.W.K. Regiment kindly gave their services, rendering an excellent programme of music during the afternoon; and having tea under the trees on a glorious June day was exceedingly popular and enjoyable. These small functions have become quite a popular feature, for, besides being profitable, they provide an excellent opportunity for the public to see over the hospital, which never fails to bring one or two new supporters into the fold.

### RAILWAY MEN AND KING EDWARD VII.'S HOSPITAL, CARDIFF.

The employees of the Great Western Railway Locomotive Department, Cardiff, have determined to maintain a bed in the Cardiff Hospital by a yearly contribution of at least 50 guineas, and the plate over the bed was unveiled on Monday afternoon last by Sir William James Thomas, whose services to this hospital and to suffering humanity in the Principality form one of the features of recent philanthropic work. Monday's ceremony was of a private character, confined as it was to a number of the employees of the locomotive department and several of the wounded soldiers at present accommodated in the hospital. These two sections of men were glad to have the opportunity of a chat, and the soldiers were quite as interested as the railway men in the proceedings. The hospital was represented by General Lee and Colonel Bruce Vaughan, who welcomed this new effort but indicated the grave needs that yet remained to be met. Colonel Vaughan reminded those present that in 1908 an appeal was started for £30,000 to add a new wing, and since that time they had not only built a new wing, but they had built a pathological block also, and spent on other adjuncts to the new wing at least £45,000, and they had added at least £90,000 to their capital. They really wanted in the institution another new wing. They wanted beds for tuberculous patients, extra wards for ophthalmic purposes, an extra ward for maternity purposes, and another operating theatre, so that people would not have to wait for operations when they came to the institution. Last, but not least, they wanted a church, so that everyone should be able to worship. The bed was named the "Simpson" bed, after Mr. Simpson, the divisional locomotive superintendent, a compliment which was gracefully acknowledged by Mr. Simpson, who was present and expressed the thanks of the men. The services of Mr. I. Johns, one of the foremen, were especially acknowledged, as it was mainly due to his initiative and spade-work that the scheme was brought to a successful issue. With Mr. Johns are associated Mr. Hodges, his fellow-representative on the hospital board, Mr. Plummer, the secretary, and Mr. Harry Russell, the treasurer of the fund. The ceremony appropriately concluded by the soldiers and railwaymen uniting to sing "God save the King," at the happy suggestion of Mr. Evans, one of the railwaymen, who remarked that they at home were also doing their bit to further the efforts of the soldiers to defeat the enemy of freedom. By their generosity the men have doubled their former contribution at a time when eighty of their number are absent with the Colours.

### AN ARMY DOCTOR WHO PREFERRED SUICIDE TO PROMOTION.

Keen disappointment at being unable to follow his own inclination and remain a private in the Army was the cause for the suicide of Hugh Evans, a temporary lieutenant in the Royal Army Medical Corps. He first enlisted as a private in the 17th Royal Fusiliers, and when it was discovered by the authorities that he was a physician and surgeon, and there was a shortage of medical men, efforts were made to induce him to accept the appointment of medical officer to his own regiment. He was a motor driver, and had made many friends amongst his comrades, and consequently he thought it was neither fair to them or to him that he should be appointed medical officer to the battalion. He refused a commission from the War Office, who, however, sent him to the Herbert Hospital at Woolwich, where he took his life. In a note which he left behind, Lieut. Evans said it might interest practitioners to learn that one night he took six grains of morphine sulphate, which had very little effect, and that the following night he increased the dose to twelve grains, but after five hours he awoke; so that he resorted to surgery, and, inflicting a wound in the groin, died from hæmorrhage. It was evident from letters left behind that it was Lieut. Evans's desire that he should have remained a private in the regiment in which he enlisted, and the removal from the barrack to the mess room did not meet with his approbation. Hence the curious reason why he took his life at the early age of twenty-six years.

### GERMAN SUPPRESSION OF THE BELGIAN RED CROSS.

At the request of the Belgian Legation in London the official Press Bureau has published a Note describing the circumstances in which the German Governor-General of the occupied provinces of Belgium has decreed the dissolution of the Belgian Red Cross Central Committee. This decree has authorised the taking over of the funds and archives of the Red Cross Society by the officer who has been delegated by the General to administer the work of the institution. Military force was employed to carry the decree into effect, and the whole forms a series of proceedings which are characterised in the Note as "an act of arbitrary violence which nothing can justify, and an injury to the work of the Red Cross which the Belgian Government cannot pass over without a protest. It is further pointed out that the Belgian Red Cross Society, which was founded in 1864, was officially recognised both by the authorities and by other Red Cross Societies. The scope of its action is defined in its articles, of which the first states its object to be "in time of war to aid the sanitary staff of the army and to succour all victims of war." The German General demanded its help for an institution called "Aid and Protection for Working Women," which he proposed to found. When the Central Committee at Brussels declined to give help, on the ground that the work proposed lay outside the objects defined in its statutes—an action which was approved by the International Red Cross Committee at Geneva—the General issued the decree of dissolution. The International Committee and the Belgian Government has solemnly protested against this arbitrary measure, in which international law has been violated, and which amounts in fact to a robbery of Red Cross funds.

### A RECORD COLLECTION FOR A RECORD WORK.

This year Hospital Sunday is being observed under more momentous circumstances than have occurred since its foundation, but it should be an encouraging thought to the hospital world and to the clergy that the brightest spot in our present anxieties is the magnificent part which the voluntary hospitals have played. The work which they are doing has surpassed all records, and we cannot but believe that the public, which has maintained so generously the work of the Red Cross Societies, will provide an equally record collection on Hospital Sunday. It cannot be denied that the Hospital Sunday collection is being held under circumstances which, from its point of view, are mainly unique in the fact that the public is taking a profounder interest in our hospitals, and is better informed concerning them, than it has ever been before. Their great work for the wounded has been the cause of this awakening, and only defective management could account for a tame response to the Hospital Sunday appeal. We await the result of the collection, then, in the confident hope of it surpassing previous totals; for our hospitals are in the public confidence and gratitude as they have never been before.

### THE RATING OF HOSPITALS OCCUPIED BY WOUNDED.

The war has not altogether distracted attention from the perennial problem of the assessment of hospital buildings for rating purposes. It has, indeed, brought forward a new aspect of the question owing to the fact that many hospital buildings, being occupied by soldiers, may claim to be War Office buildings. Such buildings the overseers as a rule do not charge for rating purposes, and the question therefore arises whether hospital buildings occupied by the wounded are to be regarded as War Office buildings for rating purposes. Before considering what the rule should be, it is necessary to point out that practical difficulties stand in the way of claiming as War Office buildings hospital blocks in which only some part is occupied by wounded. Certain hospitals have approached the military authorities for information on this matter, and the reply received is to the effect that the question of rating hospital buildings occupied by soldiers rests entirely with the overseers, who, as stated, do not as a rule charge on War Office buildings as such. It would seem, then, that if any relief on assessment is to be obtained united action on the part of hospital authorities is desirable, and probably their position would be most cogently represented if the matter were referred to the British Hospitals Association. The Association having secured a practical agreement as to the plan on the general lines of which hospital managers in receipt of the wounded propose to claim relief, could then, in view of the unprecedented services rendered by the hospitals, hardly fail to secure respectful consideration from the local authorities.

### ITALY AND THE DRUG MARKET.

Business in the drug market continues good, the demand in several departments being very active, while there is no sign of alteration in the advancing tendency of the prices of those drugs which are directly influenced by the war. Since last week's Note was written there has been another sharp advance in the price of quicksilver; this is mainly due to two causes—namely, the prohibition of the export of the metal from Italy and the large demand from France and Russia. The consequence of this advance is that calomel, corrosive sublimate, and the other salts of mercury are dearer. The future of this market is uncertain, but so far as can be foreseen there is at any rate little likelihood of any substantial reduction in value in the immediate future. Italian produce generally is naturally creating interest, and the course of the market for tartaric and citric acids, cream of tartar, essence of lemon, orris root, and olive oil should be carefully watched. In cases where the question of replenishing stocks of these articles is an urgent one, it might not be inadvisable to make purchases at an early moment. The Norwegian cod-fishing season is now practically terminated, and the yield of cod-liver oil is about 9 or 10 per cent. less than that of last season; at the moment the price of the oil has an upward tendency, but this is not very pronounced, and certainly not sufficient to alarm prospective buyers. Among articles which are dearer are aloin, preparations of antimony, butyl chloral hydrate, chloral hydrate, iron and quinine citrate, liquid extract of male fern, potassium permanganate, resorcin, salicylic acid, strychnine and thymol. The price of quinine still has an upward tendency, and the present might not be an unfavourable moment to replenish stocks.

